# Tonic ATP-mediated growth suppression in peripheral nerve glia requires arrestin-PP2 and is evaded in NF1

**DOI:** 10.1186/s40478-018-0635-9

**Published:** 2018-11-23

**Authors:** Robert A. Coover, Tabitha E. Healy, Li Guo, Katherine E. Chaney, Robert F. Hennigan, Craig S. Thomson, Lindsey E. Aschbacher-Smith, Michael P. Jankowski, Nancy Ratner

**Affiliations:** 1 Division of Experimental Hematology and Cancer Biology, Cincinnati Children’s Hospital, 3333 Burnet Ave., ML 7017, Cincinnati, OH 45229 USA; 2 Division of Pain Management, Department of Anesthesia, Cincinnati Children’s Hospital, Cincinnati, OH 45229 USA; 3 Department of Pediatrics, University of Cincinnati, Cincinnati, OH 45229 USA

**Keywords:** Glia, Schwann, PP2A, AKT, Purinergic, Neurofibromatosis, P2Y2, Arrestin, ATP

## Abstract

Normal Schwann cells (SCs) are quiescent in adult nerves, when ATP is released from the nerve in an activity dependent manner. We find that suppressing nerve activity in adult nerves causes SC to enter the cell cycle. In vitro*,* ATP activates the SC G-protein coupled receptor (GPCR) P2Y2. Downstream of P2Y2, β-arrestin-mediated signaling results in PP2-mediated de-phosphorylation of AKT, and PP2 activity is required for SC growth suppression. *NF1* deficient SC show reduced growth suppression by ATP, and are resistant to the effects of β-arrestin-mediated signaling, including PP2-mediated de-phosphorylation of AKT. In patients with the disorder Neurofibromatosis type 1, *NF1* mutant SCs proliferate and form SC tumors called neurofibromas. Elevating ATP levels in vivo reduced neurofibroma cell proliferation. Thus, the low proliferation characteristic of differentiated adult peripheral nerve may require ongoing, nerve activity-dependent, ATP. Additionally, we identify a mechanism through which *NF1* SCs may evade growth suppression in nerve tumors.

**Electronic supplementary material:**

The online version of this article (10.1186/s40478-018-0635-9) contains supplementary material, which is available to authorized users.

## Introduction

Schwann cells (SCs) are the supporting glial cells of the peripheral nervous system (PNS). In the adult nerve, specialized cellular domains maintain normal function of the nervous system via bi-directional SC-neuron communication [69]. During nerve development dynamic communication between SC and the neurons with which they associate influences SC-based axon sorting, SC proliferation, and SC differentiation [22, 27, 76]. For example, the developmental proliferation of SC precursors and immature SCs is driven by axonal neuregulin [34]. Perinatal Sox10-positive SCs associated with neuronal axons > 1 μm differentiate, elaborating myelin sheaths that increase axonal conduction velocity. As perinatal SCs differentiate into myelin-forming cells or non-myelinating cells associated with smaller axons, they exit the cell cycle. Perinatal SC proliferation and expression of myelination markers such as myelin basic protein (MBP) are mutually exclusive [3, 76, 88]. In adult nerves only 0.4% of Schwann cells incorporate markers of S-phase in a 24 h period [11, 42].

ATP is released by many types of cells, including neurons. ATP release occurs via vesicular release, maxi-anion channels, and/or through gap junctions [32]. Stevens and Fields showed that neurons firing action potentials release ATP, activating SC purinergic receptors, reducing developmental SC division, and correlating with expression of the differentiation (early myelination) marker O4 [23, 79, 80]. In SCs, gap junctions account for ATP release in this setting [61]. Although proliferation is low in normal nerves, proliferation can occur in differentiated SC after injury. In vivo, proliferation of SC increases 20–50 fold three days after nerve injury [11, 42]. In vivo nerve injury also reduces purinergic signaling, while exogenously supplied ATP prevents sequellae of nerve transection including myelin degradation [76]. Lysosome exocytosis has been proposed to account for ATP release in this setting [36]. These studies imply that nerve associated glial cells might be subject to active growth suppression.

The purine base ATP and its breakdown products ADP, AMP, and adenosine, are ligands of purinergic G-protein coupled receptors (GPCR). Activation of an uncharacterized P2 purinergic receptor (P2R) decreased proliferation in cultured SCs [79]. After binding ligand, most GPCRs signal through recruitment of heterotrimeric small g-proteins. P2Y2 is a candidate GPCR in the control of SC ATP response; P2Y2 is the major receptor responsible for Gq-mediated calcium signaling in SCs [32], and Gq was implicated in purinergic suppression of SC proliferation during development [80]. The P2Y2 receptor also couples to the small Go protein in an integrin dependent manner, activating Rac1 [4], which has been linked to anti-proliferative effects in neural crest cells and their maturing progeny, including SC [26]. SCs express mRNA encoding each of the four adenosine receptors, and respond to ADP via P2y1 and adenosine via A2a receptors in culture [79, 80]. Other studies show expression of P2x7, P2x3, P2x4, P2y4, P2y6, and/or P2y11 in satellite cells surrounding peripheral neuronal cell bodies and/or Schwann cells [9, 10, 20, 44, 60]. Of note, the P2x7 purinergic receptor is required for peripheral nerve myelination [21]. The repertoire of purinergic receptors expressed by glia suggests that purinergic signaling downstream of specific receptors may be tuned to relay specific information.

β-arrestins were identified as GPCR heterotrimeric small G-protein signal-terminating proteins. It is now known that, after binding to receptors and terminating GPCR-mediated events, β-arrestins also act as scaffolding proteins that bring together signaling complexes [14, 45]. This occurs as endocytic vesicles are internalized from the plasma membrane; for the P2Y2 receptor this can occur in association with caveolin-1 and/or clathrin [53]. Signaling events known to use arrestin scaffolds include MAPK pathway activation and modulation of PP2A phosphatase activity [64, 68]. As tools to study g-protein independent β-arrestin-dependent signaling have become available, significant contributions of the arrestins are increasingly appreciated [43].

Evasion of growth suppression is a hallmark of cancer [30]. Plexiform neurofibromas occur in 30–50% of Neurofibromatosis Type 1 (NF1) patients [56, 63, 66]. In these benign peripheral nerve tumors, SCs harbor biallelic loss-of-function mutations in the *NF1* tumor suppressor gene [31]. *NF1* encodes neurofibromin, which inactivates the RAS signaling pathway via its GTPase activating protein (GAP) domain, so that loss of NF1 function increases RAS-MAPK pathway activity [70]. How loss of NF1 alters GPCR signaling in SC remains unclear, because neurofibromin has been implicated in GPCR binding [13], Ras dependent activation of atypical PKCζ [1], and Ras-independent control of cAMP production [29], any of which may be relevant. Whether NF1 loss contributes to evasion of growth suppression is not studied. We find that SC proliferation is modulated by ATP-dependent β-arrestin/PP2A signaling, and that *NF1−/−* SCs evade the growth suppressive effects of ATP.

## Results

### Activity-dependent ATP mediates the growth suppression in non-myelinating and myelinating SCs

**Fig. 1:**
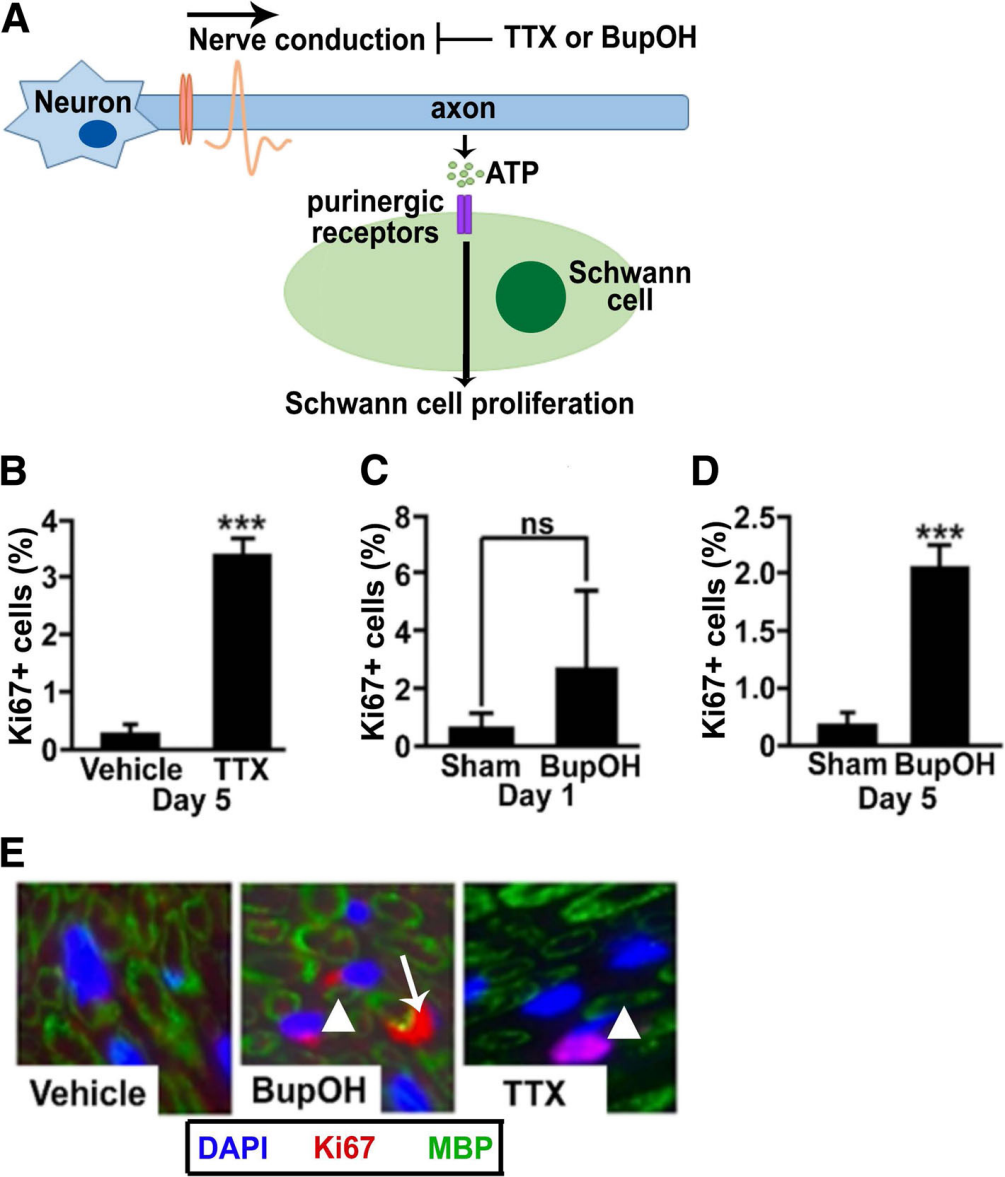
Nerve conduction controls Schwann cell quiescence in adult nerve. (**a**) Nerve electrical conduction correlates with axonal release of ATP. TTX and BupOH inhibit nerve conduction-mediated ATP release. (**b**) At Day 5, TTX-blocked WT adult sciatic nerves contained increased proliferating (Ki67+) cells (*n* = 7/group, *p* < 0.001). (**c**) No significant increase in Ki67+ cells was observed at 24 h of BupOH treatment (ns; *n* = 5/group), while (**d**) at 5 days of BupOH administration Ki67+ cells increased (*n* = 6/group, p < 0.001). (**e**) Cross sections of BupOH and TTX treated sciatic nerves were labeled with anti-myelin basic protein (MBP; green) and Ki67 (red). Cell nuclei are blue. Some Ki67+ nuclei are adjacent to MBP+ myelin sheaths (white arrow), while others were not (arrowheads)

We set out to test the hypothesis that nerve activity, via ATP, is relevant for SC growth suppression in WT nerve in vivo (Fig. 1a). To block nerve activity, we first used tetrodotoxin (TTX, n-7 mice), a selective blocker of voltage-gated sodium channels, applied by insertion of a micro capillary under the epineurium. To complement this approach, we packed Bupivacaine hydroxide (BupOH *n* = 6 mice) powder, which blocks sodium influx into neurons, along the nerve in other cohorts. Each manipulation decreased nerve function at 1 and 5 days after surgery as assessed by reduced or eliminated responsiveness to mechanical probing of the hind paw in naive animals (Additional file 1: Figure S1A, B). TTX-induced nerve block correlated with a significant 7-fold increase in Ki67+ cells (marking cells in G1, s or G2/M phases of the cell cycle) at 5 days after nerve block, versus vehicle controls (Fig. 1b). In contrast, TTX does not induce SC proliferation in culture [87]. We next examined two times points after BupOH powder application. Proliferation was non-significantly increased at one day (*P* > 0.05, Fig. 1c); however, by 5 days post treatment, proliferation had increased similarly to TTX mediated nerve block (*P* < 0.001, Fig. 1d). Ki67+; MBP+ associated cells accounted for many of the cells after TTX or BupOH-mediated inhibition of nerve activity (Fig. 1e, f). This suggests normal neuronal activity suppresses SC proliferation in vivo.

**Fig. 2:**
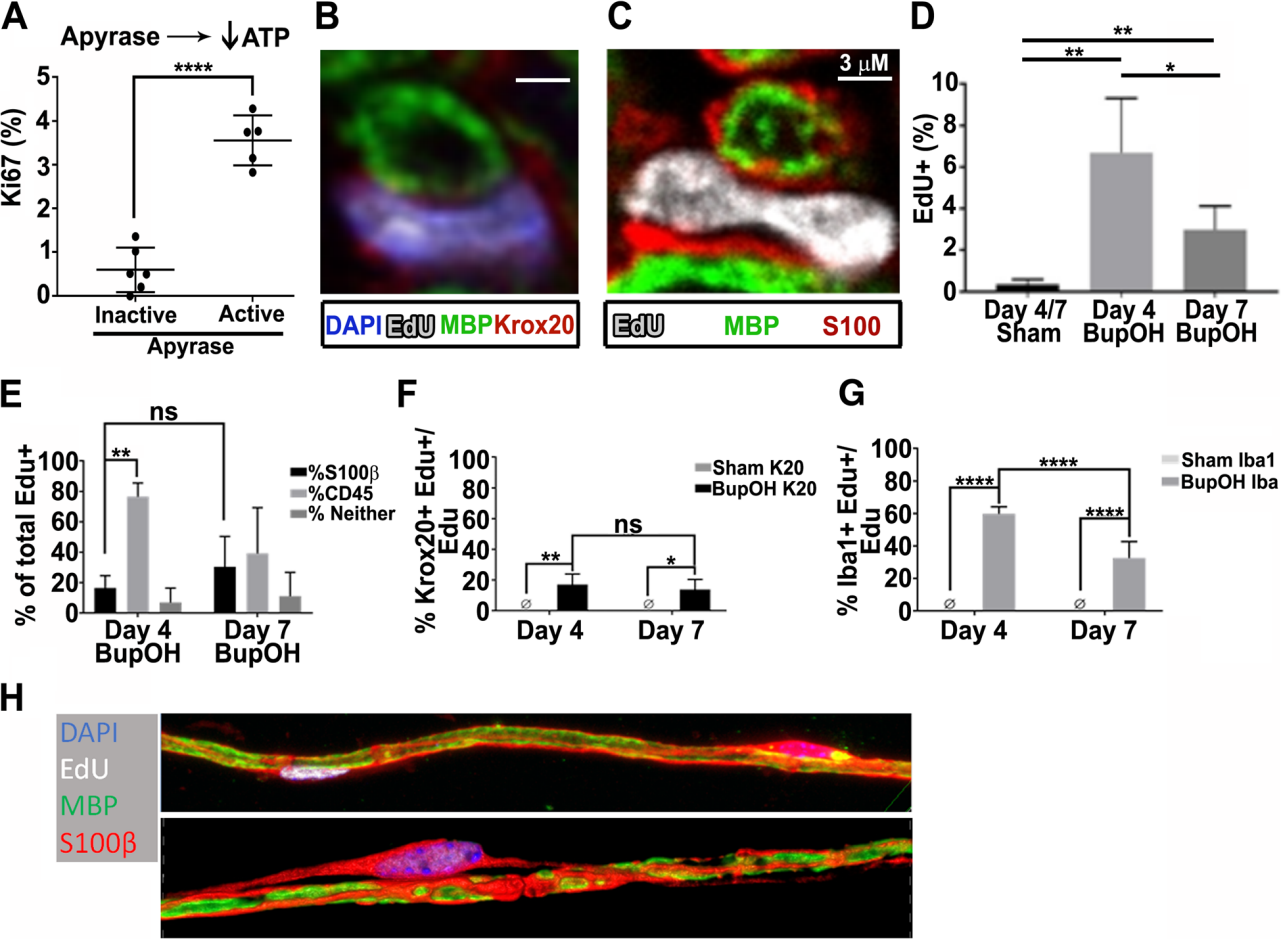
ATP is required to suppress proliferation of mature SC. (**a**) Apyrase, which degrades ATP, was administered every 4 h for 36 h IM. This resulted in increased Ki67+ cells compared to inactivated apyrase controls (n = 5active/6inactive *P* < 0.0001). (**b**) In tissue sections, EdU+ Krox20+ cells were present after Apyrase treatment, and many were associated with MBP+ myelin sheaths (**b, c**) and S100+ myelinating Schwann cell cytoplasm (scale bar = 3 μm) (**c**). In **c**, the EdU+ SC nucleus (white) adjacent to S100+ SC cytoplasm, appears to be in telophase. **(d)** Animals treated twice daily with EdU during 4 days of BupOH exposure and analyzed on Day 4 showed increased EdU+ cells over sham (*n* = 4/group < 0.01). Animals from this cohort that were sacrificed 3 days after the final dose of EdU (Day 7) showed fewer EdU+ cells (*p* < 0.05). **(e)** Broad identification of the total EdU counts reflected from the experiments represented as a percentage of total EdU. **(f)** Percentage of total EdU+ cells that co labeled with Krox20. **(g)** Percentage of total Edu + cells that co labeled with Iba1. **(h)** Confocal images of teased nerves from BupOH treated mice, top shows an EdU+ cell closely associated with an MBP+ myelin sheath and S100+ cytoplasm; bottom shows an EdU+;S100β + cell apparently separating from an adjacent MBP+ fiber

To test whether the bioactive molecule(s) responsible for in vivo entry of cells into the cell cycle is the purinergic agonist ATP, we injected wild type adult mice intramuscularly with apyrase, an enzyme which degrades ATP (*n* = 5). Heat inactivated apyrase lost activity (Additional file 1: Figure S1C), and served as a negative control. After 36 h of repetitive apyrase injection into muscle surrounding the sciatic nerve. Numbers of Ki67+ nerve associated cells significantly increased over nerves from animals injected with heat-inactivated apyrase (Fig. 2a). Thus, reducing extracellular ATP by exposure to apyrase, like blocking nerve activity to reduce extracellular ATP, promotes cell cycling. This result supports the idea that it is ATP itself, versus one of its breakdown products, which suppresses nerve cell entry into the cell cycle.

To test if SCs divide, and do not simply undergo DNA repair and/or arrest after entry into S-phase, we marked cycling cells in apyrase-treated animals during the last 20 h of apyrase exposure with the S-phase marker EdU. We then analyzed nuclear morphology in longitudinal sciatic nerve sections. Some EdU+ nuclei were Krox20+ and adjacent to MBP+ myelin sheaths (Fig. 2b). Confirming that many EdU+ cells are associated with myelinated fibers, Edu + nuclei often showed anti-S100β marked SC cytoplasm surrounding MBP+ myelin sheaths, and some of these cells showed nuclear features of dividing cells in telophase (Fig. 2c). This analysis supports the idea that ATP contributes to the characteristic low proliferation of SCs in normal peripheral nerve.

To test if cells that enter the cell cycle SC persist, we administered EdU twice daily four days (Day 4) in mice treated with BupOH (or sham surgery, *n* = 4/group). Some mice were sacrificed at Day 4. Other sham and BupOH-treated mice were maintained for 3 additional days without EdU treatment (Day 7), at which time BupOH treated mice had returned to normal sensory responses in the von Frey hair test (Additional file 1: Figure S1D). At Day 4 and Day 7, sciatic nerves from BupOH treated mice were analyzed in tissue sections. Significant increases in numbers of EdU+ cells were present at Day 4 but were significantly by Day 7 (Fig. 2d). Many proliferating cells at both time points were CD45+ (Fig. 2e), the majority of which were Iba1+ cells (macrophages), accounting for 60% of EdU+ nuclei. These EdU+; Iba1+ cells decreased significantly by day 7 (Fig. 2g). In contrast, the number of EdU+; Krox20+ myelinating SCs remained constant between Day 4 (17%) and Day 7 (14%) (Fig. 2f). Overall, we identified 94% of the EdU+ cells at Day 4 (17% Krox20+ SC, 77% CD45+ hematopoietic), and 69% at day 7 (30% Krox20+ SC, 39% CD45+ hematopoietic); remaining cells are likely to include non-myelinating SC. Importantly, some EdU+ nuclei remained associated with intact myelin sheaths at Day 7 (Fig. 2h. bottom), but others (EdU+ nuclei with S100β + cytoplasm) appeared to detaching from adjacent MBP+;S100+ myelin sheaths (Fig. 2h. top).

### Stimulation of the P2Y2 receptor mediates growth suppressive effects in wild type Schwann cells

**Fig. 3:**
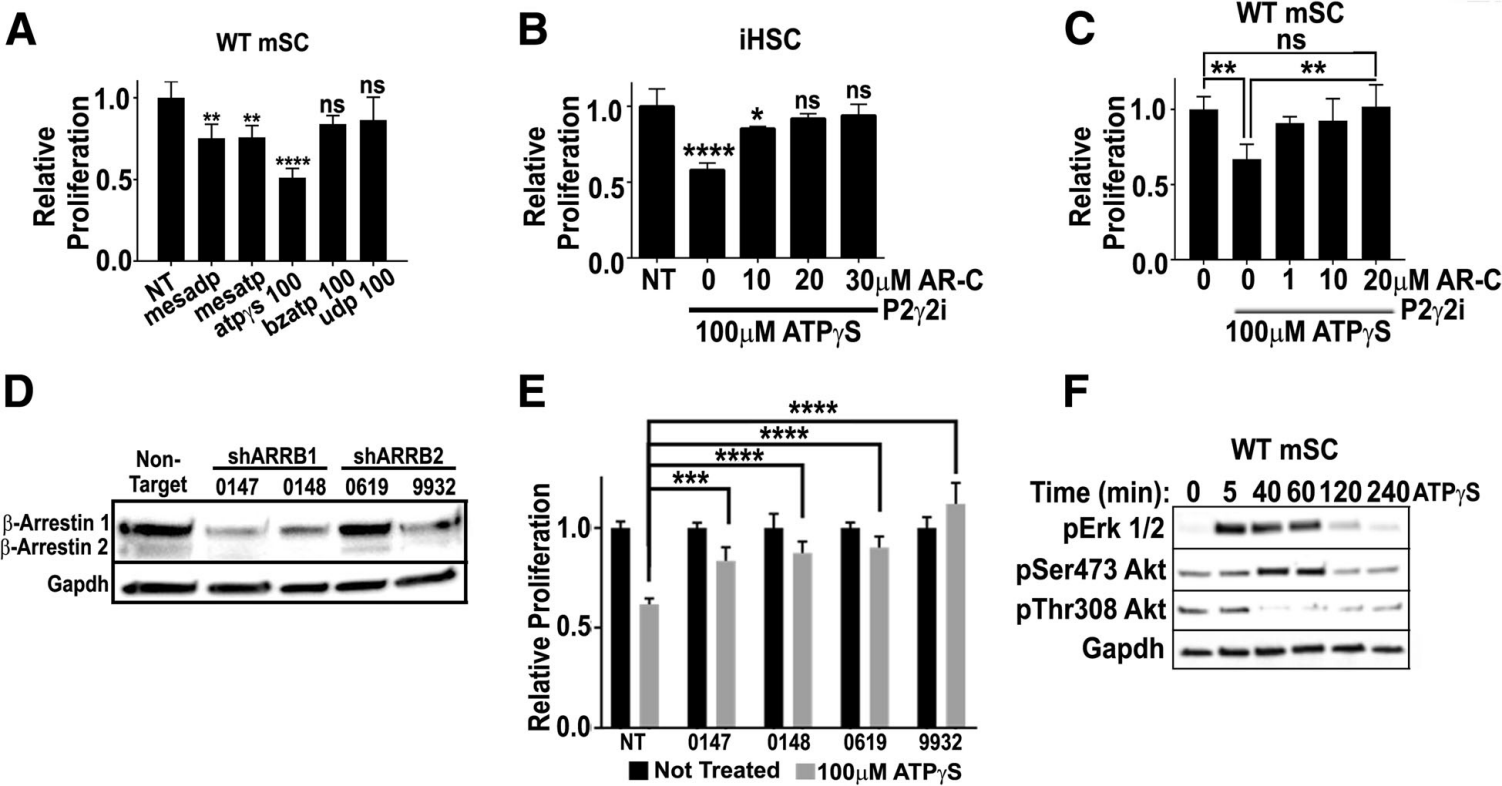
ATP-P2Y2 suppresses SC proliferation by modulating PP2A activity. **(a)** WT mSC were treated with non-hydrolysable purine analogues; ATPγS had the most significant effect on SC growth (*p* < 0.0001). **(b)** iHSC growth was suppressed by ATPγS and rescued by the P2Y2 antagonist AR-C 118925XX (AR-C). **(c)** WT mSC growth suppression by ATPγS was rescued by AR-C. **(d)** Western blot confirms reduced ARRB1 and ARRB2 protein by shRNA knockdown. **(e)** iHSCs treated with shRNA *ARRB1* and 2 shown reduced growth suppression in response to ATPγS. **(f)** Western blots of WT mSCs lysates after ATPγS treatment show increases in pERK 1/2 and pSer473 Akt. A decrease in pThr308 AKT was observed by 40 min

We tested purine derivatives for growth suppressive effects on cultured SC stimulated by β-neuregulin, a major SC growth factor. Several purinergic receptors are expressed in SCs, and many receptors can be distinguished by response to agonists and antagonists [9, 21, 82]. In mouse embryonic primary SC (mSC) and in immortalized human SC (iHSC) the non-hydrolysable ATP analog ATPγS significantly blocked neuregulin-stimulated SC proliferation (Fig. 3a, b). Relative proliferation was defined as the difference in numbers of nuclei from Day 3 to Day 0.5, normalized to vehicle treated controls. To define the receptor(s) responsible for SC growth suppression we screened receptor agonists in mSC (Fig. 3a). Neither BzATP, which activates P2X receptors, nor UDP, which activates P2Y6 and 14 receptors, caused growth suppression. The agonists 2MeSADP and 2MeSATP, which activate P2Y1, P2Y2, and other purinergic receptors [83, 85] each only partially blocked neuregulin-stimulated SC proliferation. This implicates the P2Y family of receptors in growth suppression.

To identify the relevant receptor, we tested the ability of receptor antagonists to block neuregulin-stimulated proliferation in mSC. The selective P2Y1 antagonist MRS2179 was ineffective (Additional file 2: Figure S2A). Importantly, the highly selective P2Y2 antagonist AR-C 118925XX [67] rescued the growth suppressive effects of ATPγS in eSC and also in iHSC (Fig. 3b, c), suggesting that P2Y2 is the ATP receptor in SC that mediates growth suppression in SC. Consistent with previously published results in vivo [32], genetic knockdown of P2y2 prevented proliferation of neuregulin-stimulated mSC, as monitored by Cyquant assay or by Cyclin D expression, possibly due to an additional requirement for basal P2y2 signaling in SC (Additional file 2: Figure S2B).

### P2Y2 suppresses Schwann cell proliferation in a Beta-Arrestin dependent manner

Purinergic receptors signal through associated cytoplasmic small g-proteins: G_s_, G_q_, G_i_, and/or G_12/13_ We initially analyzed G_q_-driven Ca^2+^ signaling, because it was suggested that purinergic suppression of SC proliferation might be G_q_-mediated [80] and because others have found that the P2y2 receptor is largely responsible for SC calcium signaling [32]. Gq leads to activation of phospholipase C (PLC) and increased levels of intracellular calcium. As predicted by these earlier studies, ATP, and UTP, an alternative ligand for the P2y2 receptor, increased intracellular calcium levels in SC (Additional file 2: Figure S2C). However, blocking PLC activity failed to block growth suppression (Additional file 2: Figure S2D). Therefore, we investigated another event that occurs downstream of P2y2, G_o/i_ dependent activation of Rac1 [4, 75]. Consistent with P2y2 receptor activity stimulating Rac activation, Rac1-GTP was elevated by ATP or UTP in mSC (Additional file 2: Figure S2E). However, Rac-1 seemed unlikely to entirely account for P2Y2-mediated growth suppressive effects, given that inhibition of major Rac1-mediated growth signals (Pak1 inhibition or Jnk inhibition) affected growth suppression (Additional file 2: Figure S2F, G).

In light of this negative data, we wondered if ATP-dependent growth suppressive effects in SC might be heterotrimeric G-protein-independent, and involve arrestin complexes. To test this idea, we induced genetic loss of *ARRB1* or *ARRB*2, and found that each of two shRNAs targeting *ARRB1* or *ARRB*2 significantly rescued the growth suppressive effects of ATP in iHSCs (Fig. 3d, e). We also monitored changes associated with downstream effects of β-arrestin signaling. To do this, we stimulated mSC with ATPγS, isolated samples at the indicated time points, and monitored pERK and pAKT (Thr308 and Ser473) (image representative of data from 3 independent experiments). Akt is partially activated by phosphorylation of T308 in its kinase domain; its full activation requires phosphorylation of S473 [49]. Western blotting showed a transient increase in pERK (Fig. 3f) on ATPγS stimulation, and a more delayed increase in pAKTSer473, which fell below basal levels by 2 h. In contrast, Thr308 phosphorylation of AKT decreased by 40 min., and remained low (Fig. 3f). Quantification is shown in Additional file 1: Figure S1E and 1F.

**Fig. 4:**
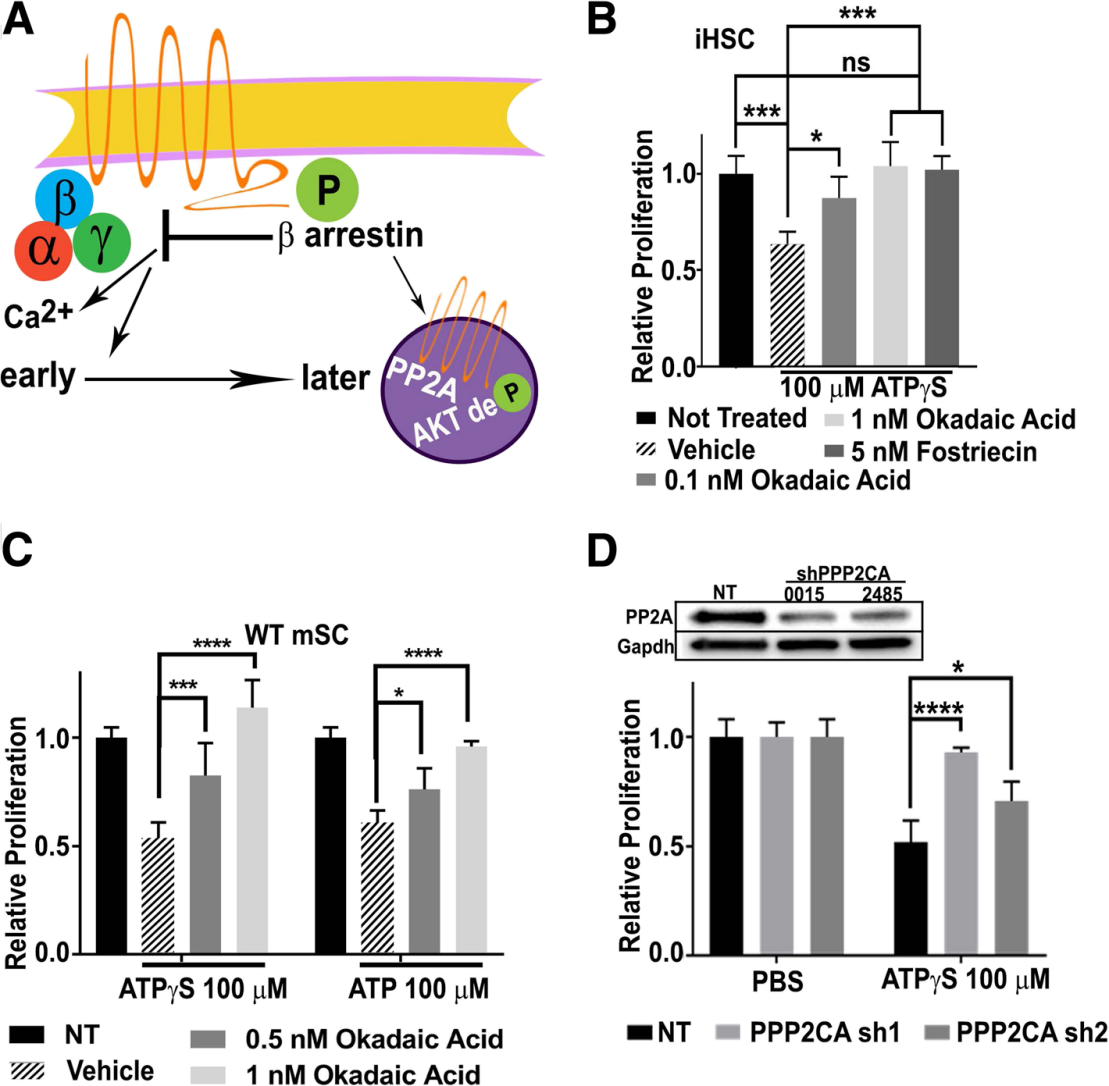
ATP requires β-arrestins to suppress SC proliferation. **(a)** Schematic representation of known G-protein-dependent calcium signaling and delayed G-protein-independent arrestin signaling. **(b)** Growth with PP2A inhibitors okadaic acid or Forstrecin rescue the anti-proliferative effects of ATPγS. **(c)** Growth suppression of WT mSCs by ATP or ATPγS was also rescued by okadaic acid. **(d)** iHSCs treated with shRNA to PPP2CA show reduced growth suppression in response to ATPγS

PP2A is a phosphatase that de-phosphorylates Akt at Thr308 and interacts with β-arrestin subsequent to GPCR signaling (Fig. 4a). We hypothesized that PP2A might be responsible for the reduced levels of P-AktThr308 on ATPγS stimulation, and be relevant in SC growth suppression. To test this, we treated iHSCs with ATPγS in the presence or absence of the PP1/PP2A inhibitor okadaic acid or with the selective PP2A inhibitor Forstreicin. Each antagonist rescued the growth suppressive effects of ATPγS in iHSC (Fig. 4b). We confirmed the rescue of ATP and ATPγS growth suppressive effects with okadaic acid in mSC (Fig. 4c). Substantiating these pharmacologic results, genetic loss of *PPP2CA*, a catalytic subunit of the PP2A phosphatase complex, significantly rescued the growth suppressive effects of ATP in iHSCs (Fig. 4d). However, while blocking AKT with MK-2206 or with the pan-Akt inhibitor Ipatasertib in mSCs potently blocked growth of SC, these treatments were significantly more potent than ATP (Additional file 2: Figure S2H). Based on these results, we suggest that stimulation of P2y2 followed by β-arrestin-mediated PP2a-stimulated de-phosphorylation (inactivation) of Akt results in growth suppression, and that Akt likely has additional effects in SC.

### ATP administration in neurofibroma-bearing *Nf1fl/fl;DhhCre* mice reduces cell proliferation and tumor formation

**Fig. 5:**
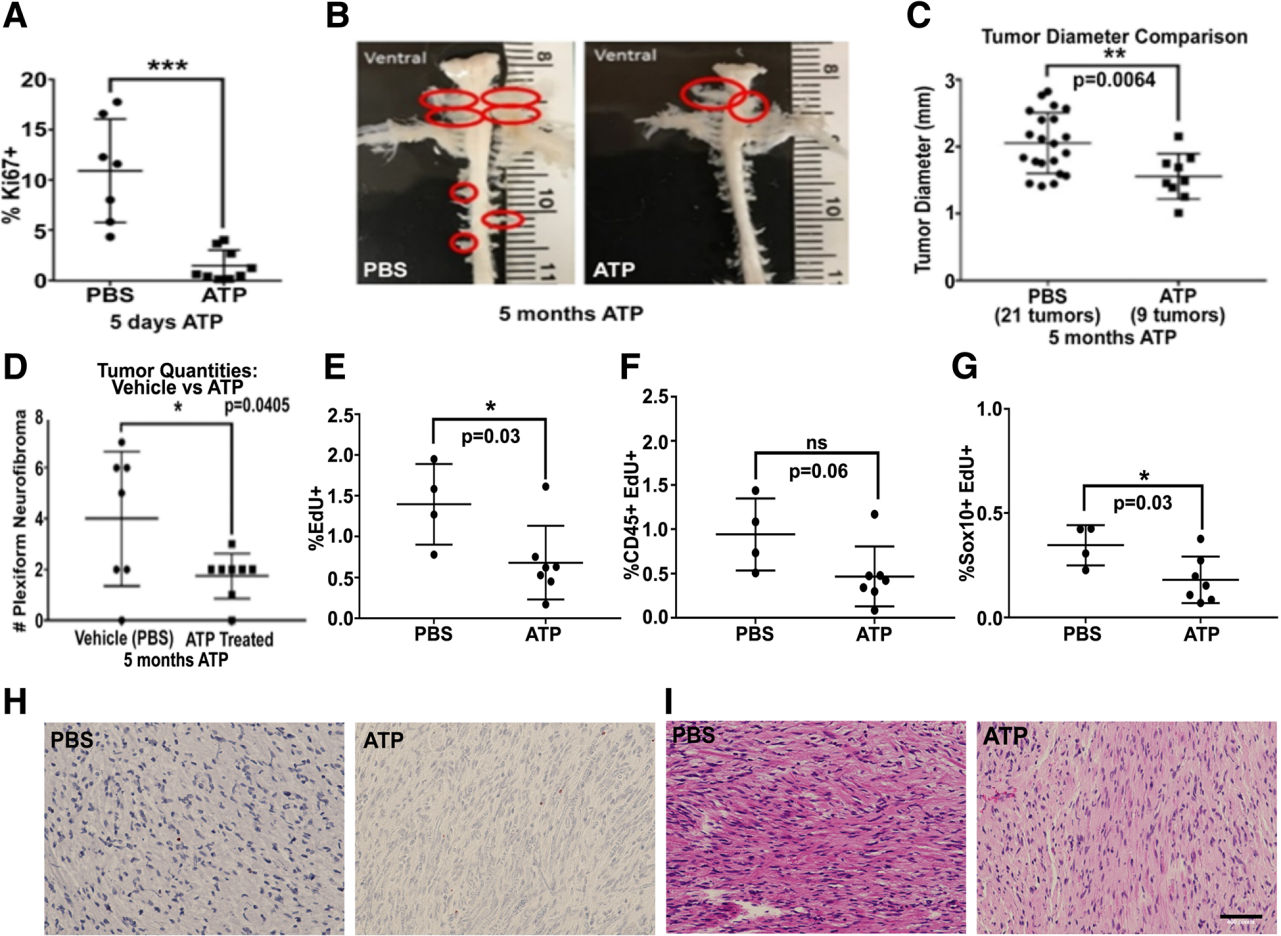
ATP is growth suppressive in neurofibroma. **(a)** Daily administration of ATP (1 mg/g/day; I.P.) for 5d reduced cell proliferation. **(b)** ATP (50 mg/kg/day; I.P.) was administered from 1 to 6 mo. of age. Representative dissections of GEM-spinal cords with attached nerves and tumors in vehicle (PBS) and ATP-treated (ATP, right) littermate. Tumors are highlighted with red circles. **(c)** The diameter of tumors, measured at the widest portion parallel to the spinal cord, was reduced in the ATP treated versus vehicle (*p* = 0.0064). **(d)** Tumor number was also reduced (*p* = 0.0405). **(e)** Total percentage of EdU+ nuclei in PBS or ATP treated tumor bearing mice. **(f)** Proportion of EdU+ cells that co-labeled with the hematopoietic marker CD45. **(g)** Proportion of EdU+ cells that co-labeled with the SC marker Sox10. **(h)** Representative images from TUNEL assay, no difference observed. **(i)** Representative H&E staining of tumors from each cohort

We hypothesized that SCs in tumors might fail to respond to ATP-mediated growth suppression, so that increasing ATP levels in vivo might reduce cell proliferation observed in neurofibromas. We tested this idea in *Nf1*-loss driven tumors. In *Nf1fl/fl;DhhCre* mice, mice form tumors by 4 mo. of age, and SC proliferation is elevated versus normal nerve [89]. Adult tumor-bearing mice (7 mo.) were treated for 5d with ATP (1 mg/g; i.p.). At this high dose, as in WT nerve, numbers of Ki67+ cells were reduced (Fig. 5a). Mice were next treated with ATP (i.p 50 mg/kg/day), beginning at 1 mo. of age. Five months later, mice were administered EdU to obtain label cells in S-phase, and then perfusion fixed. Spinal cords with attached nerve roots were analyzed (Fig. 5b) for tumor number and size. ATP treated mice showed a significant decrease in the average size of neurofibromas (*p* = 0.0064) Fig. 5c) and the overall numbers of tumors/mouse decreased (*p* = 0.04; Fig. 5d). Sections from the tumors were immune-labelled. EdU+ cells were detected in the PBS treated mice and their numbers were significantly reduced in the ATP-treated cohort (*p* = 0.03 Fig. 5e). Cell type analysis revealed that the majority of EdU+ cells were CD45+ hematopoietic cells, but the EdU+;CD45+ cells in the treatment groups did not differ significantly (*p* = 0.06 Fig. 5f). There was. However, a significant reduction in the percentage of Sox10+;EdU+ SCs in the treated cohort (p = 0.03 Fig. 5g). Cellular apoptosis was not affected (Fig. 5h, Tunnel Assay). No difference in tumor morphology was observed in H&E stained tissue sections (Fig. 5i).

### *Nf1* mutant Schwann cells are resistant to ATP mediated growth suppression

**Fig. 6:**
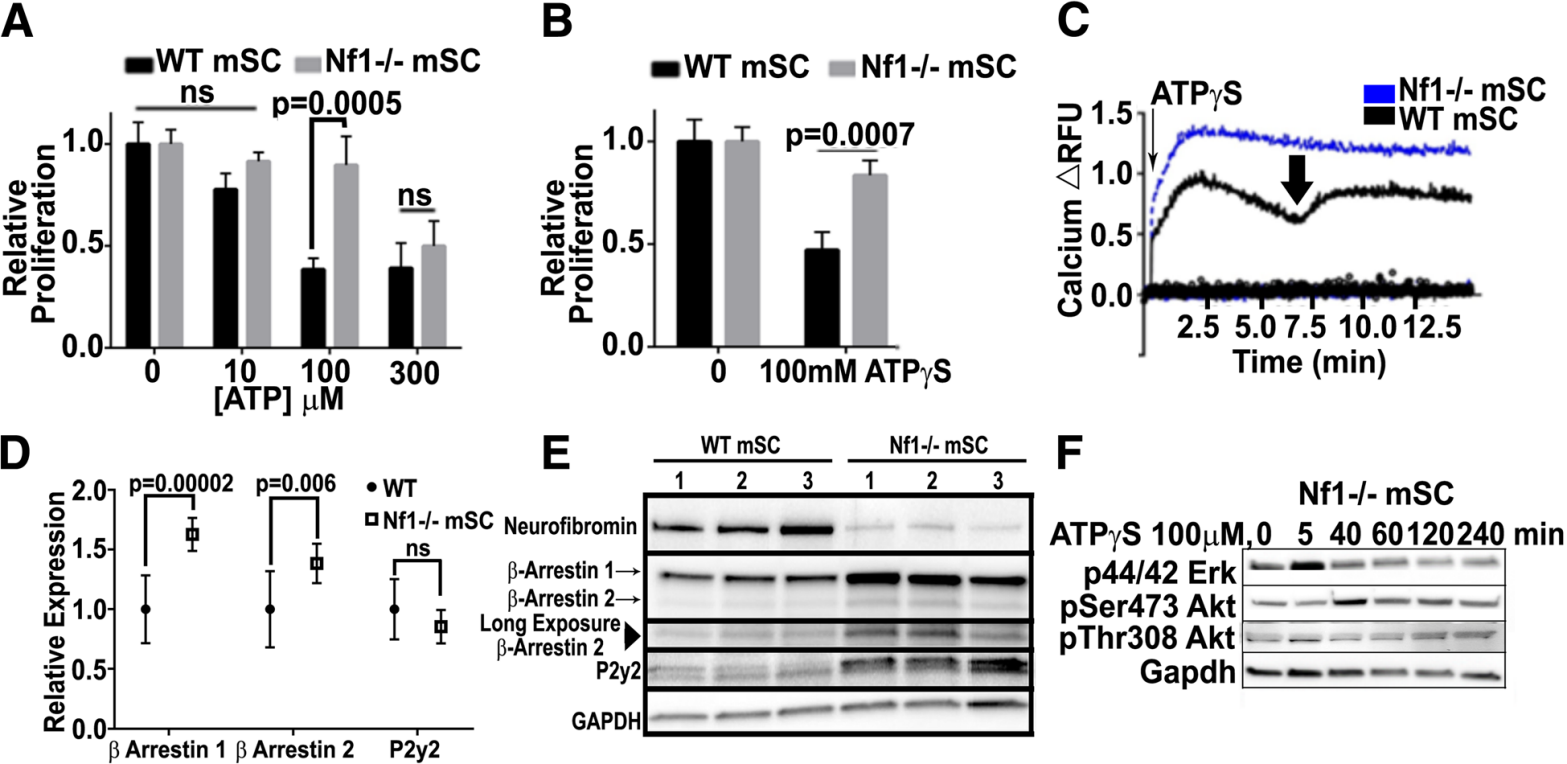
*Nf1* deficient SCs are resistant to ATP-dependent growth suppression via arrestins. **(a)** ATP (100 μM) suppresses WT mSC proliferation; *Nf1 −/−* mSCs are resistant (*p* = 0.0005). **(b)** Non-hydrolyzable ATPγS shows that differential growth suppression in WT versus *Nf1−/−* mSCs is due to ATP, not breakdown products (*p* = 0.0007). **(c)** Calcium signaling in response to ATPγS differs in WT versus *Nf1−/−* mSCs. *Nf1−/−* mSCs (blue line) lack the dip in calcium at ~ 7 min which is characteristic in WT mSCs (black line, arrow). **(d)** qRTPCR analysis of the arrestins and P2Y2 between littermate matched pairs (*n* = 3/3), both arrestins were upregulated in the *Nf1−/−* setting; however, P2y2 RNA levels were unchanged. **(e)** Western blot analysis of arrestin and P2y2 levels in WT and KO mSCs, littermate matched pairs (n = 3/3) **(f)** After ATPγS treatment, western blot in *Nf1−/−* mSCs show increases in pERK 1/2 and pSer473 Akt at early time points, similar to but reduced from WT mSCs. No decrease in pThr308 Akt was observed

While supporting the hypothesis that ATP mediates growth suppression, this in vivo test did not distinguish between effects of ATP and its breakdown products ADP/AMP/adenosine. To distinguish effects of ATP from effects of its breakdown products, we used an in vitro system, mSC lacking *Nf1* in comparison to isogenic wild type controls. A 3-day exposure to 100 μM ATP or to ATPγS reduced the growth of wild type SC, but not *Nf1−/−* mSCs (Fig. 6a, and b). Enhanced degradation of extracellular ATP by cell surface ectonucleotidases might explain reduced response to ATP but not to ATPγS, a non-hydrolyzable analogue of ATP. Higher concentrations of ATP did not further suppress WT growth. Notably, however, increasing the concentration of ATP to 300 μM was able to suppress growth in *Nf1−/−* mSCs (Fig. 6a).

We tested if β-arrestin-mediated signaling events are altered in *Nf1* mutant mSCs. While wild type and *NF1* mutant cells released Ca^2+^ from intracellular stores, Ca^2+^ transiently decreased in wild type cells before rising again (Fig. 6c), caused by arrestin-mediated arrest of GPCR signaling. This transient decrease failed to occur in *Nf1−/−* mSC, suggesting that β-arrestin signaling is reduced in the absence of *Nf1* (Fig. 6c)*.* Reduced P2y2 or arrestin might cause reduced response to ATP, but P2Y2 mRNA levels were similar in cells of both genotypes, and β-arrestin mRNAs were increased (Fig. 6d), and western blot analysis demonstrated increases in both arrestins and in P2y2 expression in *Nf1−/−* mSC (from 3 individual embryos versus WT mSC; Fig. 6e).

As shown above (Fig. 3g), in WT mSC cells exposure to ATPγS substantially increases pERK and pSer473AKT and pThr308AKT are reduced. In contrast, correlating with the evasion of growth suppression in *Nf1−/−* SC*, Nf1−/−* mSC stimulated with ATPγS increased pERK and pAKTSer473 modestly, and pThr308AKT was not reduced (Fig. 6f). As in WT mSCs, blocking AKT with MK-2206 or Ipatasertib potently blocked growth of *Nf1 −/−* mSCs (Additional file 2: Figure S2H).

**Fig. 7:**
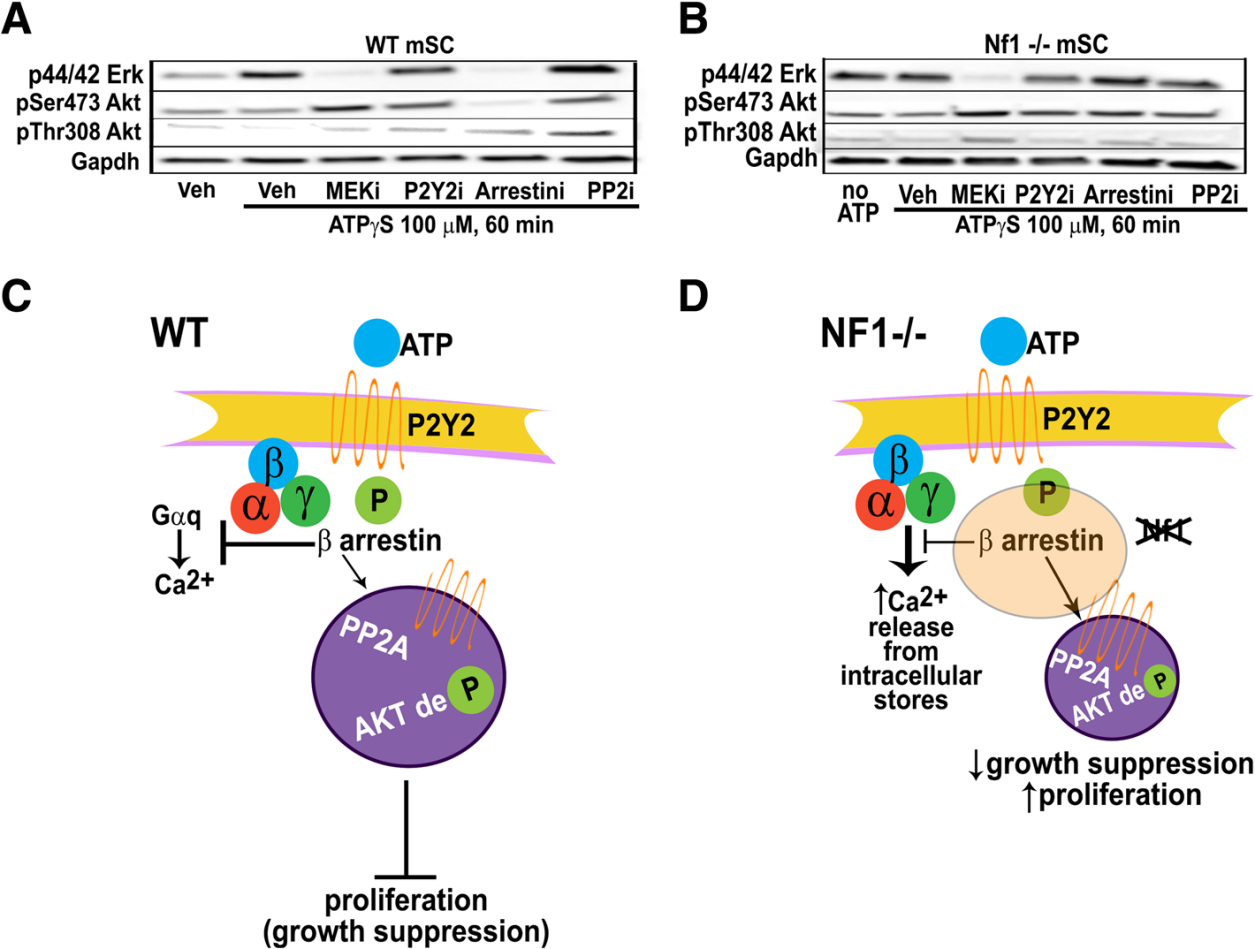
Model of ATP-dependent growth suppression in normal and *Nf1* deficient SC. **(a**) Western blot from ATPγS-treated wt mSCs or **(b)** *Nf1 −/−* mSCs (1 h) with vehicle (veh; PBS) or inhibitors (Meki = PD0325901901, P2y2i = AR-C, Arrestini = barbadin, PP2i = Okadaic acid). *Nf1 −/−* mSCs fail to decrease pERK or pAkt in response to barbadin. **(c)** ATP binds to the P2y2 receptor, causing phosphorylation and recruitment of β-arrestin(s). The β-arrestin(s) form complexes; one results in the activation of Erk. A second complex contains PP2A, which de-phosphorylates Akt, correlating with growth suppression. **(D)** When *Nf1* is inactivated ATP no longer potently suppresses SC growth. Signaling at the level of the P2Y2 receptor occurs normally, as evidenced by increased calcium on ATP stimulation. *NF1−/−* SC do not show the transient decrease in calcium characteristic of β-arrestin mediated suppression of G-protein-mediated signaling, or decrease phosphorylation of pThr308AKT

To define further the pathway causing ATPγS-stimulated changes in pERK and pAKT we added a series of inhibitors to mSC. As anticipated, in wild type SC stimulated cells with ATPγS, a MEK inhibitor blocked the increase in pERK, but did not affect P-AKT (Fig. 7a). Barbadin, an arrestin inhibitor [7], blocked increases in both pERK and pAktSer473 downstream of ATP stimulation. Barbadin also prevented the ATP-stimulated de-phosphorylation of Akt at pThr308, as did a P2Y2 antagonist and a PP2 inhibitor. Thus, activation of Erk and pAktSer473 in SCs require arrestin and Mek, while the inactivation of Akt at pThr308 requires arrestin and PP2A (Fig. 7a, and c). The ATP-stimulated increase in pERK was unaffected by P2y2 inhibition, and may be driven through other purinergic receptors.

In *Nf1−/−* mSC, the ATPγS stimulated increase in pERK was also rescued by a MEK inhibitor, but neither the P2Y2 antagonist, the arrestin inhibitor barbadin, nor a PP2 inhibitor (which normalized pThr308AKT in WT cells), affected signaling (Fig. 7b). Thus, ATP-driven signaling in WT cells is dependent upon the arrestins and PP2 (Fig. 7c), and loss of *Nf1* SCs diminishes signaling through this pathway (Fig. 7d).

## Discussion

We show that in the normal adult peripheral nerve, SC proliferation is increased when nerve activity is blocked. When ATP is removed from nerve, either by suppressing nerve activity or when ATP is degraded by apyrase, even some highly differentiated myelin-associated SC enter the cell cycle (Figs. 1, 2). Mechanistically, ATP activates P2y2, and by arrestin-dependent signaling results in PP2 driven de-phosphorylation of Akt and growth suppression (Figs. 3, 4). Therefore, not only is the nervous system sculpted by electrical activity in developing animals, electrical activity also plays roles in the adult. Importantly, SCs lacking the *Nf1* tumor suppressor evade ATP-mediated growth suppression. Resistance to purinergic agonist is rescued in *Nf1* mutant SCs by increasing levels of purinergic stimulation in vitro, and systemic administration of ATP reduced cell proliferation in mouse neurofibroma in vivo (Figs. 5, 6). Therefore, our results are applicable to neurofibroma, tumors that can cause significant morbidity in NF1 patients.

It is important to note some caveats to interpretation of our in vivo data. First, upon stimulation with ATP, SCs release ATP [50]. Thus, the source of ATP in neurofibroma bearing mice treated with exogenous ATP may be supplemented by ATP released by other cell types. Second, in vivo, administered ATP is likely to affect both SCs and immune cells. ATP acts as a chemoattractant, and ATP breakdown products have numerous effects on immune cells in vivo [19]. This may be especially relevant in neurofibromas, in which macrophages account for 30% of cells, and other types of immune cells are present [65, 92]. Indeed, following bupivacaine exposure many proliferating cells in nerve were macrophages, which may be recruited by ATP breakdown products and/or in response secondary effects of treatment [93]. Also, while SC proliferation in nerve increased after blockade of activity, and in response to apyrase, by up to 7-fold, most SC did not enter the cell cycle. This may reflect the time required for specific SCs to exit G_o_ and enter the cell cycle, the time for which an individual SC is exposed to released ATP, and/or the amount of ATP to which a cell is exposed [51].

A seminal paper showed that stimulating neuronal activity decreases developing SC proliferation and promotes SC differentiation in vitro [79]. Our finding that apyrase treatment in vivo (causing ATP degradation) mimics effects of blocking electrical activity supports these findings, and our conclusion that mature SC are under tonic ATP-mediated growth suppression in vivo. A recent publication shows that high levels of potassium can contaminate commercially acquired apyrase, which can cause effects independent of apyrase ectonucleotidase activity [52]. We controlled for this possibility by including heat inactivated apyrase (from the same enzyme lot) and controlling buffer conditions. The rapid increase in SC proliferation we show in electrically silent adult nerves also suggests that normal nerve contains growth promoting signals that are suppressed by electrical signaling. Such positive signals may include mitogens and ATP breakdown products. For example, the ATP breakdown product adenosine enhances neuron-stimulated neuregulin-mediated SC growth in vitro, and in SC, adenosine activates MAPK signaling and cell growth [57, 79, 81].

After decreasing nerve ATP, 20% of EdU+ cells, including those with nuclear features of dividing cells, are differentiated, myelinating SC. These SCs express Krox20 and are associated with elaborate compact MBP+ myelin sheaths. In axon-SC co-culture, SCs associated with myelin sheaths also divide, after their associated axons are cut [72]. Thus, while proliferation and differentiation are considered mutually exclusive during development [34], proliferation can occur in differentiated cells in the adult nerve. In vivo, SCs associated with degenerating fibers also proliferate after nerve transection (Pelligrino et al., 1986; Clemence et al., 1989). For example, nerve transection under the L3-L5 vertebrae resulted in myelinating SC proliferation in the sciatic nerve, distal to the injury (Murinson et al., 2005). These authors suggested that cell division resulted in one daughter cell leaving the axon. Our morphological evidence is consistent with this idea, as we frequently visualized S100+ cells with Edu + nuclei detaching from adjacent S100+; MBP+ myelinating fibers. Blocking ATP release or ATP degradation resulted in division of the nuclei of differentiated myelinating SC, which might have predicted altered myelinated fiber morphology or formation of short myelin internodes, if two nuclei persisted in a single myelinating SC, but we did not find evidence of two separated nuclei with attached myelin sheaths. Inhibiting electrical activity with TTX does not cause axon degeneration or alter myelin structure, even at the electron microscope level [54], consistent with the idea that at least under some conditions that even if a SC nucleus divides, the structure of the myelin sheath and its attached nucleus does not suffer. In yeast and in *C. elegans*, early cell division results in cells of two different sizes, mediated through GPCR signaling [41]. Given the maintenance of myelin structure on decreased ATP in nerve, one possibility is that there is apical division of the nucleus with SC cytoplasm, leaving the remainder of a myelinating SC intact.

We define the pathway required for SC growth suppression by ATP as downstream of P2Y2. Our results differ from those of a study in which shRNA targeted *P2y2* in vivo, which did not affect SC proliferation. In vivo, compensation among GPCRs may occur; it will be of interest to study purinergic receptor expression in model SC systems. Growth suppression requires the highly homologous β-arrestin 1 and 2 proteins, which are ubiquitously expressed and highly conserved across species, but can play different physiological roles [64, 68]. The roles of β-arrestins in GPCR trafficking and intracellular signaling have been increasingly appreciated, and structural alterations in GPCR that alter arrestin interactions are known in detail [37, 45]. Our finding in SC are similar to those in arterial smooth muscle cells, in which reducing β-arrestin prevented P2Y2 receptor desensitization [58]. P2Y2 can interact with both arrestin proteins [58], and in iHSC, both β-arrestins may play roles downstream of ATP-P2Y2, as receptor activation occurred normally yet targeting β1 and β2 each partially rescued growth suppression. Reduced β-arrestin function, resulting from the inability to desensitize receptors and thus terminate GPCR signaling and/or changes in arrestin signaling in cells has been implicated in nervous system function and cancer [45].

PP2 plays a critical role in ATP-mediated SC growth suppression, as PP2 inhibition prevented growth suppression in SC. In addition to Okadaic acid, which inhibits PP1 and PP2A, we used fostriecin, which inhibits PP2A with an IC50 value of 1.5 nM, and high selectivity over PP1 and PP2B [86]. PP2A is a multi-subunit complex with numerous regulatory subunits, which can appear to act as a tumor suppressor or oncogene. It is now believed that reduced PP2A activity promotes cell transformation, while normal levels of PP2A activity are critical for cell survival [71].

A biochemically confirmed arrestin-PP2A-AKT complex was documented downstream of dopamine D2 receptor activation [6]; this complex mediates lithium action on behavior [5]. PP2A associates with both arrestins under multiple conditions [14, 38, 64, 90]. While blocking PP2A rescued AKT de-phosphorylation, and correlated with rescue of SC growth suppression, we cannot be certain that AKT is the relevant PP2A substrate in SC. Although PP2A is required for cell cycle exit, blocking AKT completely blocked SC growth stimulated by serum and neuregulin [24]. This result supports additional roles for AKT in SC, likely including AKT effects on SC survival and myelin sheath thickness [18, 48, 78]. PP2A, associated with arrestin has substrates in addition to AKT, including those which modulate trafficking of ion transporters [40]. These might act in SC growth suppression, alone or together with AKT.

ATP-mediated growth suppression was reduced in mouse and human SCs with inactivating mutations in the *NF1* gene. *Nf1* mutant cells showed failure of arrestin-mediated suppression of GPCR-mediated Ca2+ release and of arrestin-mediated, PP2A-dependent, de-phosphorylation of Akt. The most direct explanation of these results is that arrestin function is modulated, directly or indirectly, by neurofibromin. Neurofibromin binds directly to RAS accelerating the hydrolysis of GTP on RAS, and binds many other proteins [70]. Mutations in *NF1* cause increased SC RAS-GTP, but whether elevated RAS-GTP explains the *NF1* deficient SC escape from anti-proliferative effects of ATP remains to be determined. AKT can be activated downstream of RAS-GTP, by interaction of active RAS with PI3K isoforms [16, 25] and/or indirectly via crosstalk with the mTOR pathway (Mendoza et al., 2011). Increased levels of ATP reduced SC proliferation in the knockout setting. A likely explanation of this finding is that with increasing ATP concentration, receptors less sensitive to ATP than P2y2 are activated. For example, P2y1 is activated at high ATP concentrations, despite being largely ADP selective [83].

Recent attention has focused on RAS-RAF-MEK-ERK signaling in neurofibroma, given that MEK inhibition shrinks neurofibroma in preclinical and clinical trials [17, 35]. On ATP stimulation, the extent and duration of Erk activation were altered in *NF1* deficient SC. However, Bz-ATP, which does not cause growth suppression, correlated with altered ERK activation (not shown), suggesting that ATP activation of a receptor other than P2y2 drives Erk activation, and that SC growth suppression driven by ATP is MEK/ERK independent. In HEK293 cells, ERK activation was G-protein dependent and to some extent independent of arrestins, indicating a predominant scaffolding function of arrestins [28]. However, barbadin, which interferes endocytosis of receptors associated with arrestins by interacting with the interface between β-arrestin and the β2-adaptin subunit of the clathrin adapter protein AP2, blocks ERK activity in HEK293 cells [7]. In our study, blocking arrestin activity with barbadin prevented ERK activation, consistent with the latter study. Overall, our data demonstrates a reliance on the arrestins consistent with their established role in cell signaling.

The tumor suppressive effects of neurofibromin are of intense interest given the morbidity of NF1 disease and the large numbers of sporadic tumors now known to show *NF1* mutations [55, 74, 77]. Targeting purinergic receptors in tumors is also an active field of study [2, 8, 15], and activation of PP2A is being tested [59, 73]. Neurofibromin-regulated GPCR-arrestin signaling may contribute to the aberrant activity of a broad array of receptors, and thus a number of clinical manifestations [64]. Overall, peripheral nerve SC show tonic inhibition of cell proliferation, which we have shown requires P2Y2 and arrestins, and is modulated by *NF1*. Therefore, restoration of purinergic signaling to effect growth suppression may augment current approaches for therapy in *NF1* mutant nerve tumors.

## Materials and methods

### Mice

All animal experiments were conducted in accordance with institutional procedures under approved protocols reviewed by the Institutional Animal Care and Use Committee of the Cincinnati Children’s Hospital. Wild type C57Bl/6 mice were from Jackson Laboratory and were used at 2–4 months old. *DhhCre; Nf1flox****/****flox* mice were maintained on a predominantly C57Bl/6 background; their genotyping by PCR has been described [89]. Dhh-Cre mice were maintained on the C57Bl/6 background [33]. Mice of both sexes were used for all experiments.

### In vivo sciatic nerve block

Mice were anesthetized with isoflurane and right sciatic nerves were exposed. Bupivacaine hydroxide (BupOH) powder prepared as described [94] was loosely packed along the nerve and the wound closed with sutures [91]. For nerve block with TTX, a modified version of Li et al. was employed [47]. Borosilicate glass capillaries were pulled on a microelectrode puller and fire polished to create a rounded tip with an outer diameter of approximately 50 μm and an internal diameter of approximately 10 μm. The back ends of microcapillaries were fire-polished to produce rounded ends with a slight opening to allow back-filling with TTX or control citrate buffer, then filled with bone wax and silicone gel to seal the ends and to prevent leakage. Microcapillaries were surgically inserted under the epineurium into the space between the diverging sural, tibial, and common peroneal nerves [47, 54]. Wounds were closed with 6.0 silk sutures. Controls for these experiments were citrate buffer filled micro capillaries for TTX, or sham surgery in which the nerve was exposed and sutured but no drug was applied for BupOH.

### ATP and apyrase injections

ATP (Sigma; 1 mg/g) or PBS (vehicle) of injected daily (i.p.) for 5 days in 2 mo. old adult mice. Mice were sacrificed 1 or 5 days later, and sciatic nerves dissected and fixed. Other mice were administered 50 mg/kg ATP or PBS daily (i.p.) for 5 mos. In apyrase experiments, apyrase (Sigma catalog# A6535-1kU) was reconstituted at 100 units/mL sterile PBS. As a control, this solution was heat inactivated at 95 °C for 5 min.; loss of activity was confirmed by a modification of the Celltiter Glo assay (Additional file 1: Figure S1C). We administered 50 μL (5 U) apyrase intramuscularly (I.M.), every 4 h. (for 36 h.), into the left hind leg, rotating clockwise around the nerve to reduce the number of times we injected each site [19, 62, 84]. Beginning 16 h. after the first injection, EdU was injected I.P. (50 mg/kg every 4 h. Mice were sacrificed and sciatic nerves dissected, and fixed for analysis.

### Sensory assays

To assess sensory function we performed von Frey filament stimulation of the plantar surface of hindpaws with 2 – 10 g filaments. Animals were placed in a clear Plexiglas container with a steel mesh bottom for at least 30 min prior to testing. We determined the minimum gram force to which 100% of WT mice elicited a withdrawal response (6 g). For mice that underwent BupOH or TTX nerve block, baseline measurements were made in the same mice that underwent surgical manipulations, 1 h. prior to capillary implant or BupOH powder treatment. Mice were assessed 1 day prior to surgery and on the day of sacrifice. Mice underwent sensory testing with 5 min between trials. The average number of times the animals withdrew from the supra-threshold stimulus was determined over 3 trials and averaged.

### Teased nerves

Anaesthetized mice were trans-cardially perfusion-fixed with cold PBS, then with ~ 30 mL of 4% paraformaldehyde in phosphate buffer pH = 7.2. We dissected sciatic nerves, which were washed in ice-cold PBS for up to 48 h. After removing the endoneurium, nerve fragments were teased with needles in 100 μL PBS into individual fibers, on Superfrost microscope slides (Fisher Scientific cat# 12–550-15), air dried then frozen at − 20 °C prior to immunostaining.

### Immunohistochemistry/fluorescence

For frozen sections, OCT was removed by incubation with PBS. We permeabilized cells in ice cold MeOH for 10 min., followed by incubation in normal donkey serum (Jackson ImmunoResearch cat# 017–000-121) and 0.3% Triton-X100 (Sigma-Aldrich Cat# X100). Primary antibodies and dilutions were: Ki67 [11F6] (1:200, Biolegend cat# 151202), MBP (1:200 SCBT cat# sc-13,914), Krox20 (1:400 Abcam cat# ab43020), Sox10 (1:100 SCBT cat# sc-17,342), S100 (1:1000 Agilent Technologies Cat# Z031129–2), S100β [EP1576Y] (1:1000 Abcam cat# ab52642) All secondary antibodies were donkey anti Rat/Rabbit/Goat from Jackson ImmunoResearch, reconstituted in 50% glycerol and used at 1:250 dilution. To visualize nuclei, sections were stained with DAPI for 10 min., washed with PBS and mounted in FluoromountG (Electron Microscopy Sciences, Hatfield, PA). Images were acquired with ImageJ Acquisition software using a fluorescence microscope (Axiovert 200 M) with 10x/0.4 or 40x/0.6 objectives (Carl Zeiss, Inc.), or with NIS-Elements software using confocal microscopy (Nikon).

### Intracellular calcium assay

SCs were cultured in 96 well plates in SC media (previously described) to near confluency. A Fluo-4 direct calcium assay kit was used according to manufacturer’s protocol, with 60 min. Incubations at 37 °C (1 h total prior to stimulation). Agonist (or antagonist) was added using FlexStation 3 injection system (Molecular Devices). Fluorescence was measured continuously for 15 s prior to addition of agonist, then for 5–15 min. at 480 nm/525 nm with 1 read/~ 0.5 s. Relative fluorescence was calculated as described in the Fluo-4 assay kit.

### Cell culture

Embryonic e12.5 primary mouse SCs (mSC) were isolated from dorsal root ganglia with neuronal contact in N2 medium with nerve growth factor, then removed from neurons and cultured in SC media (DMEM + 10% FBS + b-heregulin + forskolin) for 1–3 passages as described [39]. Immortalized 1λ human SCs (iHSC) and *NF1−/−* 95.11bc immortalized human SC from a neurofibroma (*NF1−/−* iHSC) were cultured in DMEM supplemented with 10% FBS as described [46].

### Cell viability assay

SC were plated at 2–3000 cells/well in 100 μL in 96 well plates in SC media (mSC), or DMEM + FBS (iHSCs). After 4 h. we added compound(s) (2 μL) to quadruplicate wells for 3 days (mSC) or 1 day (iHSC). Proliferation was measured with Cyquant (Thermo Fisher Cat# C35011) on a Molecular Devices SpectraMax utilizing the nuclei counting mode, 4 regions/well. All proliferation data is representative of at least 3 independent experiments.

### Statistics

Two-group comparisons used Student’s *t*-tests. When single agents were tested at different concentration in a single cell type, we used a one-way ANOVA with a Dunnett’s multiple comparisons test. When multiple genotypes were analyzed in a single experiment, we used a 2-way ANOVA with multiple comparisons, without matching, and correction with the Holm-Sidak test. Significance set at 95%. All data unless otherwise stated is represented as average ± SD or relative standard deviation, and was analyzed in GraphPad Prism 7.

### Plasmids, and primers

mP2y2 primers fwd 5’-CTGGTCCGCTTTGCCCGAGATG-3′ rev 5’-TATCCTGAGTCCCTGCCAAATGAGA-3′ [12]. mArrb2 fwd 5’-GCTGAAACCACACGCCACTT-3′ rev 5’-CCTGGCTTCCAGCACCATTG-3′. mArrb1 5’-GGACCCAGGACAGAGCAGAT-3′ rev 5’-GAGAAGGGAGGCCACAGCTT-3′. mbeta-actin fwd 5’-CGGTTCCGATGCCCTGAGGCTCTT-3′ rev 5’-CGTCACACTTCATGATGGAATTGA-3′. shRNA human ARRB1 CCGGAGATCTCAGTGCGCCAGTATGCTCGAGCATACTGGCGCACTGAGATCTTTTTTG, CCGGTCTGGATAAGGAGATCTATTACTCGAGTAATAGATCTCCTTATCCAGATTTTTG (TRCN0000230148 and TRCN0000230147 respectively, SigmaAldrich). shRNA human ARRB2 CCGGGCTAAATCACTAGAAGAGAAACTCGAGTTTCTCTTCTAGTGATTTAGCTTTTTTG, CCCCGGGCTAAATCACTAGAAGAGAAACTCGAGTTTCTCTTCTAGTGATTTAGCTTTTTG (TRCN0000159332 and TRCN0000280619 respectively, SigmaAldrich). shRNA human PPP2CA GTACCGGACCGGAATGTAGTAACGATTTCTCGAGAAATCGTTACTACATTCCGGTTTTTTTG, CCGGGAGGGATATAACTGGTGCCATCTCGAGATGGCACCAGTTATATCCCTCTTTTT (TRCN0000380015 and TRCN0000002485).

## Additional files

Additional file 1: **Figure S1.** A) Von Frey filament, and grip strength analysis for BupOH and TTX at days 1 and 5. B) Von Frey filament analysis for BupOH treated mice taken at day 1. C) Apyrase activity assay, inactivated apyrase (squares) did not reduce ATP levels as determined by luminescence. D) Von Frey filament analysis for mice used in the pulse chase experiments. E) Quantification of primary wt mSC western blot intensities after ATP treatment normalized to non phosphorylated forms plotted over time. F) Quantification of primary Nf1-/- mSC western blot intensities after ATP treatment normalized to non phosphorylated forms plotted over time. G) WT mSC proliferation assay, treated with two different AKT inhibitors. (PDF 2423 kb)

Additional file 2: **Figure S2.** A) Proliferation analysis of different purinergic agonists in the presence of a P2y1 antagonist (no affect). B) Proliferation of WT mSC after knockdown of P2y2 and confirmation of knockdown by Western Blot. C) Calcium assay comparison of ATP and UTP on e12.5 wt mSCs. D) Primary WT and Nf1-/- mSC proliferation assay to assess the effect of a PLC activator. E) Rac1-GTP pull down assay of WT and Nf1-/- mSCs treated with ATP or UTP. F) Proliferation Assay on primary WT mSCs examining the effects of Jnk (JNK-IN-8) or PLC (U73122) inhibition on ATP dependent growth suppression. G) Proliferation assay on primary WT mSCs examining the effects of Pak inhibition on ATP dependent growth suppression. H) Proliferation assay on primary Nf1-/- mSCs in the presence of two different AKT inhibitors. (PDF 12848 kb)
