# Supplementary figures and images for: Tonic ATP-mediated growth suppression in peripheral nerve glia requires arrestin-PP2 and is evaded in NF1
Source: Acta Neuropathol Commun. 2018 Nov 23;6:127. doi: 10.1186/s40478-018-0635-9 (PMC6251093; doi:10.1186/s40478-018-0635-9)

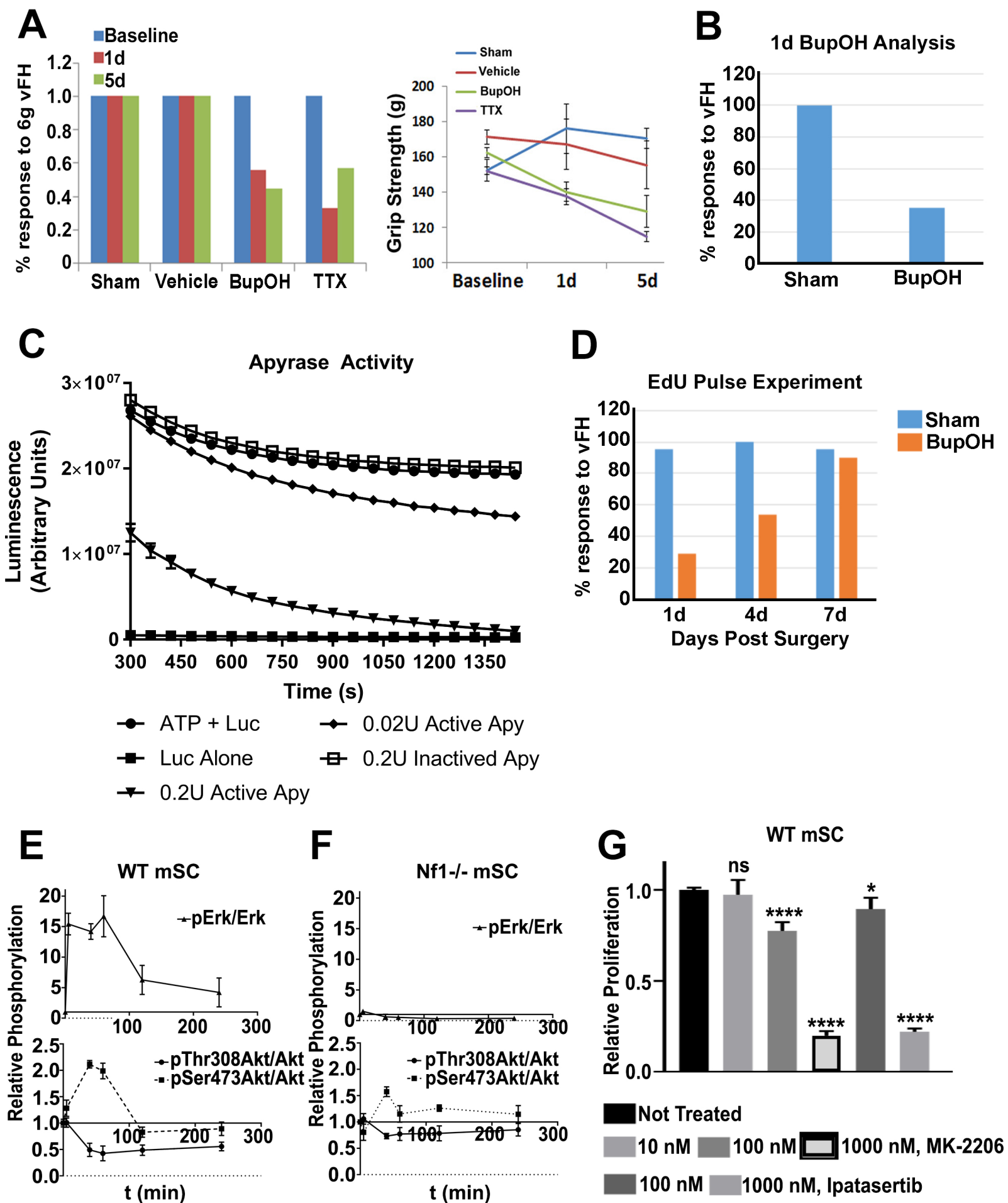

Supplement: Supplementary file 1 — Figure S1. A) Von Frey filament, and grip strength analysis for BupOH and TTX at days 1 and 5. B) Von Frey filament analysis for BupOH treated mice taken at day 1. C) Apyrase activity assay, inactivated apyrase (squares) did not reduce ATP levels as determined by luminescence. D) Von Frey filament analysis for mice used in the pulse chase experiments. E) Quantification of primary wt mSC western blot intensities after ATP treatment normalized to non phosphorylated forms plotted over time. F) Quantification of primary Nf1-/- mSC western blot intensities after ATP treatment normalized to non phosphorylated forms plotted over time. G) WT mSC proliferation assay, treated with two different AKT inhibitors. (PDF 2423 kb) [file 40478_2018_635_MOESM1_ESM.pdf]

# Supplemental 2

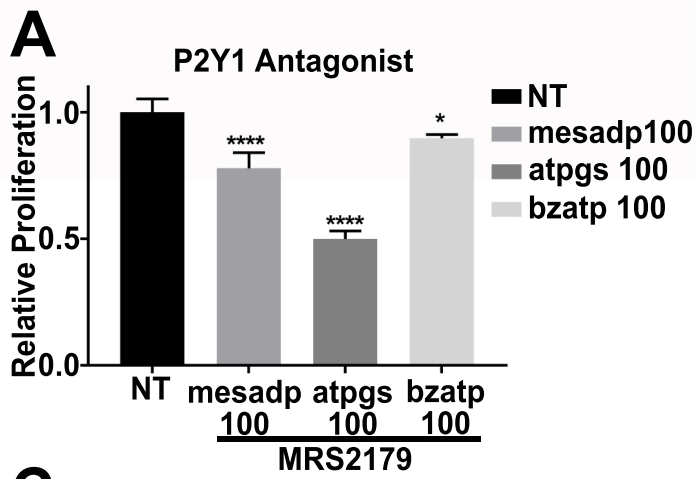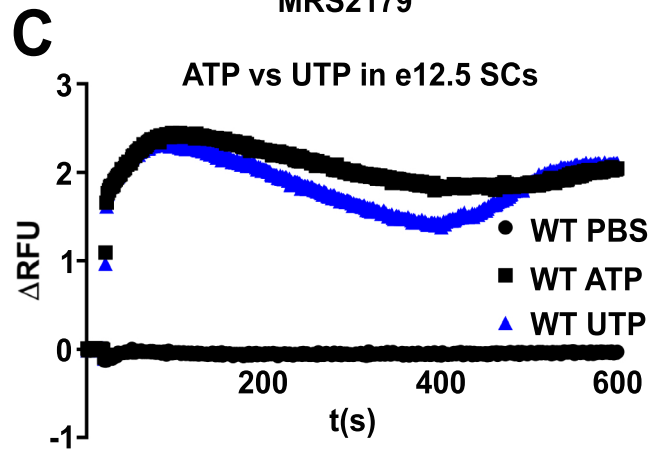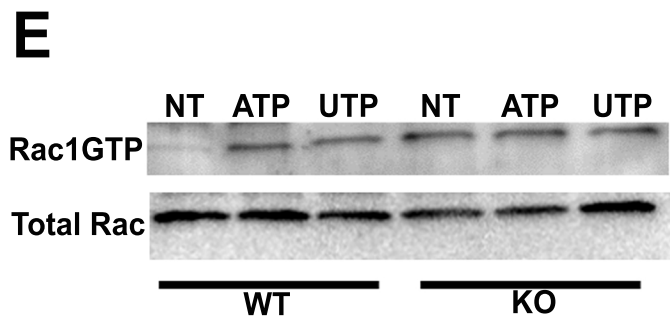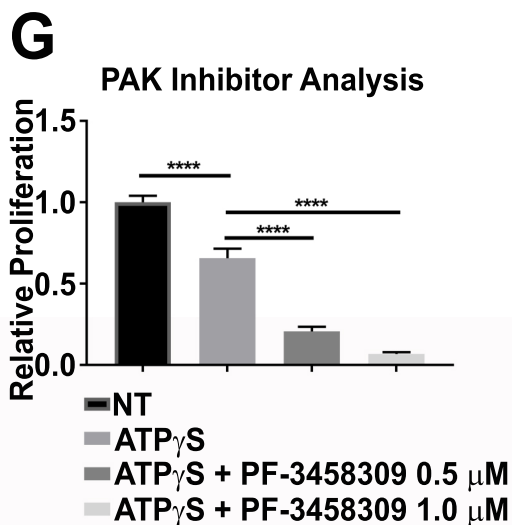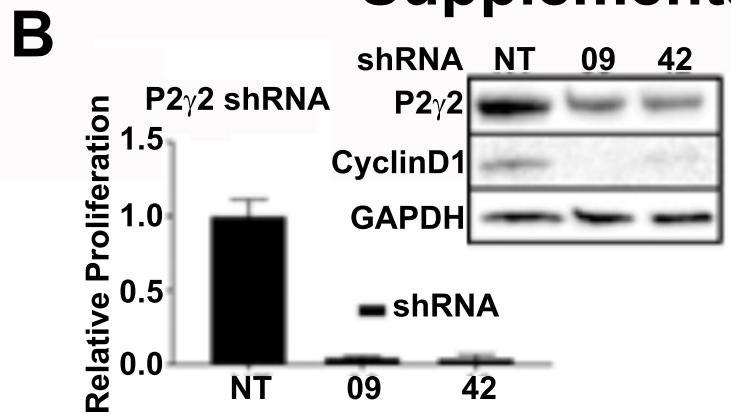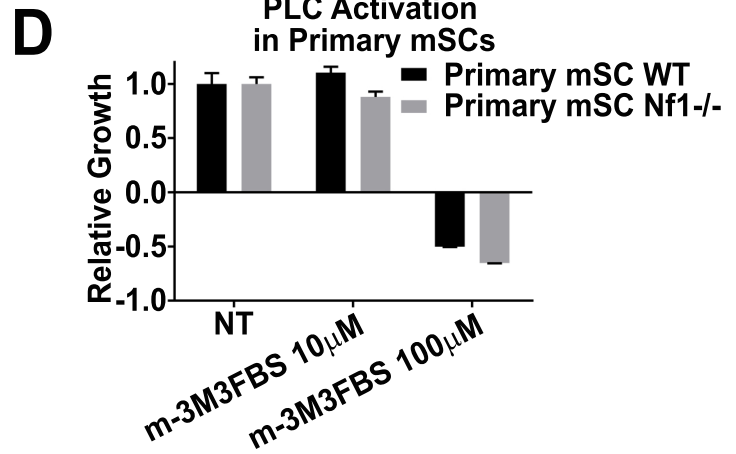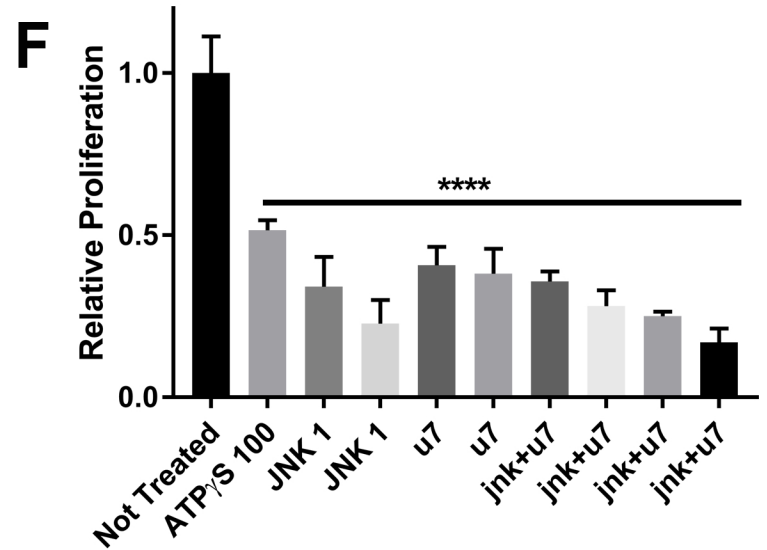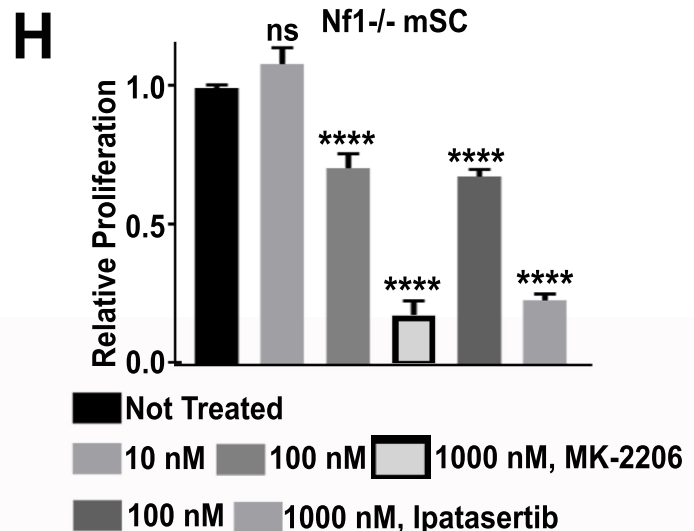

Supplement: Supplementary file 2 — Figure S2. A) Proliferation analysis of different purinergic agonists in the presence of a P2y1 antagonist (no affect). B) Proliferation of WT mSC after knockdown of P2y2 and confirmation of knockdown by Western Blot. C) Calcium assay comparison of ATP and UTP on e12.5 wt mSCs. D) Primary WT and Nf1-/- mSC proliferation assay to assess the effect of a PLC activator. E) Rac1-GTP pull down assay of WT and Nf1-/- mSCs treated with ATP or UTP. F) Proliferation Assay on primary WT mSCs examining the effects of Jnk (JNK-IN-8) or PLC (U73122) inhibition on ATP dependent growth suppression. G) Proliferation assay on primary WT mSCs examining the effects of Pak inhibition on ATP dependent growth suppression. H) Proliferation assay on primary Nf1-/- mSCs in the presence of two different AKT inhibitors. (PDF 12848 kb) [file 40478_2018_635_MOESM2_ESM.pdf]
